# Evaluation of QSAR Equations for Virtual Screening

**DOI:** 10.3390/ijms21217828

**Published:** 2020-10-22

**Authors:** Jacob Spiegel, Hanoch Senderowitz

**Affiliations:** Department of Chemistry, Bar-Ilan University, Ramat-Gan 5290002, Israel; spiegel.jacob@gmail.com

**Keywords:** Quantitative Structure Activity Relationship (QSAR) models, QSAR equations, virtual screening (VS), enrichment-based optimization, multiple linear regression (MLR), random forest (RF), support vector machine (SVM), enrichment optimizer algorithm (EOA)

## Abstract

Quantitative Structure Activity Relationship (QSAR) models can inform on the correlation between activities and structure-based molecular descriptors. This information is important for the understanding of the factors that govern molecular properties and for designing new compounds with favorable properties. Due to the large number of calculate-able descriptors and consequently, the much larger number of descriptors combinations, the derivation of QSAR models could be treated as an optimization problem. For continuous responses, metrics which are typically being optimized in this process are related to model performances on the training set, for example, $R^{2}$ and $Q_{CV}^{2}$. Similar metrics, calculated on an external set of data (e.g., $Q_{F1/F2/F3}^{2}$), are used to evaluate the performances of the final models. A common theme of these metrics is that they are context -” ignorant”. In this work we propose that QSAR models should be evaluated based on their intended usage. More specifically, we argue that QSAR models developed for Virtual Screening (VS) should be derived and evaluated using a virtual screening-aware metric, e.g., an enrichment-based metric. To demonstrate this point, we have developed 21 Multiple Linear Regression (MLR) models for seven targets (three models per target), evaluated them first on validation sets and subsequently tested their performances on two additional test sets constructed to mimic small-scale virtual screening campaigns. As expected, we found no correlation between model performances evaluated by “classical” metrics, e.g., $R^{2}$ and $Q_{F1/F2/F3}^{2}$ and the number of active compounds picked by the models from within a pool of random compounds. In particular, in some cases models with favorable $R^{2}$ and/or $Q_{F1/F2/F3}^{2}$ values were unable to pick a single active compound from within the pool whereas in other cases, models with poor $R^{2}$ and/or $Q_{F1/F2/F3}^{2}$ values performed well in the context of virtual screening. We also found no significant correlation between the number of active compounds correctly identified by the models in the training, validation and test sets. Next, we have developed a new algorithm for the derivation of MLR models by optimizing an enrichment-based metric and tested its performances on the same datasets. We found that the best models derived in this manner showed, in most cases, much more consistent results across the training, validation and test sets and outperformed the corresponding MLR models in most virtual screening tests. Finally, we demonstrated that when tested as binary classifiers, models derived for the same targets by the new algorithm outperformed Random Forest (RF) and Support Vector Machine (SVM)-based models across training/validation/test sets, in most cases. We attribute the better performances of the Enrichment Optimizer Algorithm (EOA) models in VS to better handling of inactive random compounds. Optimizing an enrichment-based metric is therefore a promising strategy for the derivation of QSAR models for classification and virtual screening.

## 1. Introduction

Quantitative Structure Activity Relationship (QSAR) analysis could be broadly defined as the application of mathematical/statistical methods in order to find an empirical relationship between dependent variables obtained for a set of objects, and independent variables which describe in some ways these objects. In the most common QSAR applications, the dependent variables are activities (defined in the broadest possible way), the objects are molecules/materials and the independent variables are structure-based molecular/materials descriptors. Over the years, QSAR analysis has been widely and successfully used in various research areas including chemistry, biology, toxicology and materials sciences in order to both analyze the factors affecting molecular properties and to design new compounds with improved properties [1,2,3,4,5,6,7].

Building predictive QSAR models requires the following steps: (1) Data collection, namely, the assembly of a large enough, well curated dataset of compounds with accurate activities, preferably measured by one source (e.g., laboratory) or at least according to the same protocol. (2) Data preparation, which typically includes structure curation and descriptors calculation and pre-processing (e.g., removal of constant, nearly constant and correlated descriptors). (3) Model derivation employing one of numerous machine learning approaches. The modeling process begins with a training set (modeling set), and proceeds by performing regression based or classification-based analysis to construct a model of activity as a function of the descriptors. Machine learning techniques are mostly used in this area, because they can deal with very complex relationships between structures (as perceived by the descriptors) and activities [8]. (4) Model validation. Whenever possible, QSAR models should be validated on an external set (termed validation set or test set) [9]. An external set can be obtained by splitting the input dataset prior to the model development phase or by obtaining additional data [10,11]. When the number of samples in the input dataset is too small to allow for a reasonably large test set, models are usually evaluated using cross validation. Thus, the most common metrics for evaluating QSAR equations developed for continuous responses are $R^{2}$ (for the training set) and $Q_{F1/F2/F3}^{2}$ (for the external set) [12,13,14]. Classification-based models, i.e., models derived for categorized responses are evaluated by metrics derived from the confusion matrix (e.g., the Matthews Correlation Coefficient; MCC).

Due to the large number of molecular descriptors available for QSAR analysis (for example, the popular DRAGON software can calculate ~5300 descriptors [15]) the number of descriptors combinations is too large to allow for an exhaustive search for that combination that would afford the best model. As a result, the derivation of QSAR models could be treated as a single objective, multi-variables optimization problem [16]. Solving the so-called features selection problem requires an optimization engine and an objective function to be optimized. Several optimization algorithms have been used to derive QSAR models including Genetic Algorithm (GA) [17,18], Particle Swarm Optimization (PSO) [19], Iterative Stochastic Elimination (ISE) [20] and Metropolis Monte Carlo coupled with Simulated Annealing (MC/SA) [21]. As to objective functions, the most commonly used ones are model evaluation metrics calculated for the training set such as $R_{train}^{2}$, $Q_{train-CV}^{2}$, MAE (Mean Averaged Error) or the regression standard deviation.

As mentioned above, QSAR models could highlight the factors affecting molecular properties and could also be used to design new compounds with improved properties. The success of the latter task depends on the ability to transform the insight obtained from the models into new designs, which in turn requires the models to be interpretable. Alternatively, the design problem could be replaced by using QSAR models in order to perform virtual screening (VS) of large collections of commercially available compounds found in numerous databases in search for those compounds with favorable properties. Indeed, several such efforts, using QSAR models developed on both continuous and categorized responses, were reported in the literature [22,23,24].

Many QSAR models derived from continuous responses in whichever manner yet with VS in mind are still evaluated using R^2^/Q^2^-like metrics. We argue however that there is no *a priori* reason to expect QSAR models characterized by favorable R^2^/Q^2^ values to perform well in VS (and vice versa). Thus, alternative, VS-aware, metrics should be used for both model derivation and evaluation. Specifically, and borrowing from the most common methods used for VS, namely, pharmacophore modeling and docking, we propose that QSAR models for VS should be evaluates based on metrics derived from enrichment/Receiver Operating Characteristics (ROC) data.

To test this hypothesis, we focused on Multiple Linear Regression (MLR) as a common method for deriving QSAR equations. First we derived 21 MLR models for seven datasets corresponding to seven targets (5-Hydroxytryptamine Receptor 2C (5HT_2C_); Muscarinic acetylcholine receptor M2 (M2); Histamine H1 receptor (H1); human Ether-à-go-go-Related Gene (hERG); Muscarinic acetylcholine receptor M3 (M3), Dopamine receptor (D1), Alpha-2C adrenergic receptor (Alpha_2C_)), and evaluated their performances on the training set (using $R^{2}$), on an external validation set (using $Q_{F1/F2/F3}^{2}$) and finally on two additional external sets (termed test sets) designed to mimic small-scale virtual screening campaigns. In accord with our hypothesis we found little correlation between the $R^{2}$/$Q_{F1/F2/F3}^{2}$ metrics and the ability of these equations to pick up active compounds from within a collection of random (assumed to be mostly inactive) compounds. Similarly, we found poor correlation between model performances on training/validation/test sets when using a VS-aware evaluation metric.

Next, in order to obtain models where performances on training/validation sets would better inform on performances for VS, we developed an algorithm that derives QSAR models in the form of MLR equations by directly optimizing an enrichment-based function. We term this new algorithm Enrichment Optimizer Algorithm (EOA) and the resulting equations, EOA models or equations. When tested on the seven, above-described datasets, this new algorithm indeed gave more consistent results for the training, validation and test sets. Importantly, performances of the best EOA models on the test sets, which, as noted above, were constructed to mimic small-scale VS campaigns, were in almost all cases either similar to or better than performances obtained with the original MLR equations. An advantage of our new algorithm is that it can use as input binary activity data (i.e., active/inactive) rather than quantitative data as typically required by continuous response MLR equations.

Finally, since virtual screening is akin to a classification problem, we compared the performances of our new algorithm on the seven data sets with those of two of the state of art classifiers, namely, Random Forest (RF) and Support Vector Machine (SVM), obtaining overall better results across training/validation/test sets, in most cases. We attribute the better performances of the EOA models in VS to better handling of inactive random compounds.

## 2. Results

Table 1 presents the results of the MLR models obtained for the seven datasets considered in this work and Table S1 provides the actual equations. In each case, models were derived by optimizing the regression standard deviation metric in the space of the input descriptors using 10^6^ MC steps (see Methods section for more details). Model performances on the training and validation sets were evaluated using the “classical” metrics, $R^{2}$ and $Q_{F1/F2/F3}^{2}$ for the training and validation set, respectively, as well as by counting the number of active compounds found within the first *L* places (See Table 1 and Table 2 for the values of *L*) of the sorted list of the predicted *pKi* values. Enrichment factors derived from this metric using Equation (5) (see Methods section) are also provided in parenthesis (see Table 5 in the Methods section for maximal enrichment values attainable for each dataset). For the test sets, only the number of active compounds found within the first *L* places of the sorted activity list and the corresponding enrichment values are provided (since no information on the actual activities of the random compounds is available).

Analyzing the results presented in Table 1, we first note the favorable correlation between the number of active compounds identified in the training and validation sets. Correlating these numbers across all models, we find a Pearson correlation coefficient (r^2^) of 0.9, a slope of 1.2 and an intersect of −13.0. Since the validation sets constitute perfectly valid external sets (i.e., they were not used to train the models in any way), we take this to indicate that the MLR models are not over-fitted. At the same time, however we observe a large mismatch (and no numerical correlation) between model performances evaluated using $R^{2}/Q_{F1/F2/F3}^{2}$ and using the enrichment metric calculated for the test sets, in accord with our basic conjecture. Thus, all models generated for the M2, M3, D1, and Alpha_2C_ sets are characterized by favorable $R^{2}/Q_{F1/F2/F3}^{2}$ values, which are mirrored, by excellent enrichment values for both training and validation sets. Yet, only the M2 and D1 models constructed from 7 descriptors and all Alpha_2C_ models were able to identify active compounds from within test set 1 (see below for a more detailed discussion). For the H1 set we observed a similar trend as for M2, M3, and D1 with respect to performances on the training and validation sets (albeit with lower $Q_{F1/F2/F3}^{2}$ values), and even poorer performances for test set 1. For the 5HT_2C_ set, a sharp decrease from $R^{2}$ (0.58–0.70) to $Q_{F1/F2/F3}^{2}$ (−0.08–0.18) is observed, yet the corresponding decrease in enrichment values (from 43–45 to 30–32) is moderate while the enrichment for test set 1 is extremely poor. Finally, for the hERG dataset, both $R^{2}$ and $Q_{F1/F2/F3}^{2}$ values are low yet the enrichment values for the training, validation and test set 1 are excellent. Looking at the results obtained for test set 2, we first note that due to data set limitations (see Methods section), in some cases the number of active compounds is too low to attach much meaning to the results (e.g., M3, Alpha_2C_ and to a lesser extent D2). Nevertheless, we still observe poor correlation in terms of the number of retrieved active compounds between training and test sets (Pearson correlation, r^2^ = 0.16 and 0.00 for test set 1 and test set 2, respectively) and between validation and test sets (r^2^ = 0.25 and 0.00 for test set 1 and test set 2, respectively) across all models.

Table 2 presents the best (out of five repeats) corresponding results, as judged by performances on the test sets obtained with the EOA models. A compilation of all the results is provided in Tables S2–S8 (first sheet in each table) in the supplementary material. Model performances are expressed in terms of the number of active compounds found within the first *L* places of the sorted list of the predicted *pKi* values. Enrichment factors derived from this metric using Equation (5) are also provided in parenthesis. To facilitate a comparison with the MLR results (Table 1) we colored in red, yellow and green EOA models with performances on test sets poorer than, similar to or better than the corresponding MLR models.

Similar to MLR, EOA models also demonstrate good correlation between the number of active compounds identified in the training and validation sets (r^2^ = 0.85, slope = 0.9, intercept = −1.4) suggesting that the models are not over-fitted. The intercept value is smaller than that observed for the MLR models (−13.0) suggesting a better training/validation correspondence in terms of the retrieved actives. Moreover, in comparison with MLR models, EOA models show an overall better consistency across the training, validation and test sets with Pearson correlation coefficients (r^2^) between the number of active compounds retrieved in the training and test set 1 and in the validation and test set 1 of 0.37 and 0.28, respectively. Importantly, these values increase to 0.63 and 0.51 upon the removal of a single (out of 21) deviating model (M3 model with 10 descriptors). This consistency is most apparent for the M2, hERG, and two of the M3 models, is less apparent for the H1, and D1 models but is absent from the 5HT_2C_ and Alpha_2C_ models. Because of the overlap between the active compounds in the validation and test set 1 (see Method section for more details), we attribute the poorer performances on the test set to poorer handling of random (yet presumed to be inactive) compounds. Still, EOA models perform in this respect better than MLR models. Indeed, for four datasets, M2 H1, M3 and D1, the performances of the EOA models on the test set were significantly better than those of MLR while for two datasets (5HT_2C_ and hERG) both methods performed similarly. Only for the Alpha_2C_ dataset did the MLR models outperform the EOA models. Looking at the results obtained for test set 2 we first re-iterate the small number of active compounds in some of the dataset and the large discrepancy in this parameter across all sets, which hampers rigorous quantitative analyses. Nevertheless, we observe that EOA models were as good as or better than MLR models in 18 cases. It is interesting to note that in all cases (except the D1 model with 10 descriptors; see footnote of Table 2) the best EOA models for the two test sets were identical.

**Figure 1:**
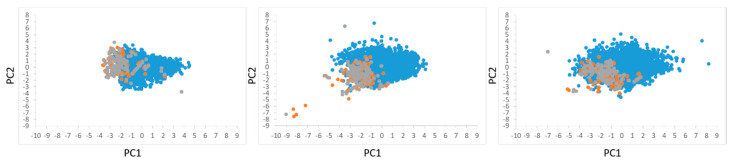
Principle Component Analysis (PCA) plots of training set (orange), validation set (grey) and test set 1 (blue) compounds in the space of the descriptors comprising the 7-descriptors model (**left**), 10-descriptors model (**middle**) and 13-descriptors model (**right**) for the M2 dataset. The first two PCs cover 49%, 42%, and 35% of the original variance for the 7-descriptors, 10-descriptors, and 13-descriptors models, respectively.

The MLR results obtained for the M2 and M3 datasets are intriguing (Table 1). Favorable $R^{2}/Q_{F1/F2/F3}^{2}$ values were obtained for all models yet only the M2 model constructed from seven descriptors was able to pick active compounds from within the pool of random compounds when run on both test sets (a single compound was also picked up from within test set 2 by the M2 model with 10 descriptors). One possible explanation for this phenomenon is that the applicability domain of the M2 model with 7 descriptors better covers the test set compounds than the applicability domains of the M2 models with 10 and 13 descriptors. To test this hypothesis, a principle component analysis (PCA) was performed on the descriptors spaces comprising the three M2 models while considering the training, validation and test set 1 and the results are presented in Figure 1. While the first two PCs cover only <50% of the original variance, it is clear that for the 7-descriptors space the average distances between the training and test set compounds are smaller than in the 10- or 13-descriptors spaces. To quantify this visual observation, we calculated the average distances between all training and test compounds in all three spaces using all PCs and found the distance in the 7-descriptors space to be statistically significantly smaller (using Student’s t-test) than the distances in both 10- and 13-descriptors spaces (4.75±1.06, 5.56±1.96 and 5.50±1.30, for the 7-, 10- and 13-descriptors space, respectively). We note, that due to the central limit theorem, a t-test could also be used for non-normal distributions provided the size of the samples is large enough which is the case here. Thus, we propose that test set 1 compounds (and test set 2 as well, since the decoy compounds of both test sets, are identical) are more within the boundaries of the applicability domain for the 7-descriptors M2 model than for the 10- and 13-descriptors models which may explain the better performances of the first model. This applicability domain-based rationalization is in fact analogous to the above-mentioned suggestions that EOA models better handle random test set compounds.

Importantly however, based on the results presented in Table 2, the EOA algorithm is less affected by whichever factors (e.g., applicability domain) which led to the poor performances of the 10- and 13-descriptors M2 MLR models in virtual screening. This may explain the overall better performances of the EOA algorithm on test sets 1 and 2.

Virtual screening is engaged with the identification of a small set of active compounds from within a large pool of random, yet presumed to be inactive compounds. Thus, this procedure could be considered as a binary classification problem (albeit performed on a highly skewed dataset). This is true even if the VS engine assigns continuous activity data to the screened compounds because in order to submit a certain number of virtual hits to biological evaluation, the continuous data should be binned into active/inactive groups by applying an activity threshold. With this in mind, we wanted to compare the performances of EOA models, this time evaluated as binary classifiers, with those of two of the state of the arts classifiers, namely, Random Forest (RF) and Support Vector Machine (SVM). For consistency, we used the same seven datasets, yet derived different training/validation/test sets, also including compounds with binary (rather than continuous) activity data (see Table 6 in the Methods section for training/validation/test sets compositions). Each set was modeled five times using different random seeds to initiate the MC/SA procedure. The results are provided in Table 3 and Table 4 for test set 1 and test set 2, respectively. To allow for a facile comparison with RF/SVM results, we colored in green the best models (as judged be performances on the test sets) from within EOA, SVM and RF.

Looking at Table 3 (test set 1), a comparison between the best EOA and RF models for the test sets suggests identical or better performances of the former in terms of enrichment and MCC for 27 and 25 models, respectively. A similar comparison between EOA and SVM suggests identical or better performances for the former in terms of enrichment and MCC for 28 and 23 models, respectively. Looking at Table 4, similar comparisons suggest identical or better performances of EOA with respect to RF in terms of enrichment and MCC for 24 and 22 models, respectively, and identical or better performances of EOA with respect to SVM in terms of enrichment and MCC for 28 and 21 models, respectively. Thus, in most cases best EOA models outperform both RF and SVM models. It is also noteworthy that in 19 cases, the same model was found to be the best one for both test 1 and test 2. Table S9 provides the components, which are used to calculate the MCC values, namely, True Positive (TP), True Negative (TN), False Positive (FP), and False Negative (FN) rates for all EOA, RF and SVM models derived in this work as well as their average values (last sheet). Based on these data, it is evident that on average, the best EOA models (averaged over test set 1 and test set 2) outperform RF models in terms of Test-TP, Test-TN, Test-FP, Test-FN, Test-MCC, and Test-enrichment and SVM models in terms of Test-TP, Test-FP, Test-MCC, and Test-enrichment. In both cases, the largest differences in favor of the EOA models are found in the Test-FP rate. This is an important parameter in virtual screening, in particular for less resource rich entities, since a high FP rate may lead to investing substantial resources in inactive compounds. A comparison of averaged values across all EOA-models with those obtained for RF suggests similar performances in terms of all parameters except that RF has a slightly better MCC. A similar comparison with SVM suggests similar performances in terms of most parameters except that SVM affords slightly better MCC values and markedly better Test-FN rates whereas EOA is slightly better in terms of enrichment.

## 3. Discussion

The last few years have witnessed the development of extremely large datasets of commercially available or virtual yet synthetically feasible drug-like molecules and materials with potentially interesting properties [25,26,27]. Virtually screening such large collections is beyond the capabilities of many computational techniques (e.g., pharmacophores, docking) and due to the enormity of the chemical/material space this gap is unlikely to be closed in the near future. Machine learning-based models on the other hand are ideally suited for large scale VS campaigns due to their speed and since in many cases they could rely on rapidly calculate-able 2-dimensional descriptors [28,29]. However, how exactly to evaluate the performances of such models for VS is still, in our view, an open question.

Evaluating QSAR models has been the subject of extensive discussions in the literature [9,12,13,14,30] with the general outcome that models are typically evaluated on external test sets using a metric that quantifies the agreement between experimental and predicted activities. Similar metrics are used for feature selection when the derivation of QSAR models is treated as an optimization problem. However, most of these discussions evolved around the selection of the appropriate dataset on which the model should be validated or on the mathematical details of the validation metrics and were less concerned with the intended usage of the resulting models.

The main message of the present work is that the development of machine learning models should explicitly take into account their intended usage and that failure to do so, may lead to misinterpreting the model performances. More specifically, we propose that machine-learning models developed with virtual screening in mind, should be derived and evaluated using a virtual screening aware metric. Importantly, we do not claim that QSAR models developed and validated by other means are not appropriate for VS. Indeed, several successful applications of such models have been reported in the literature. However, we suggest that, a priori, there is no reason to expect that QSAR models characterized by favorable values of the most common performance-evaluating metrics (e.g., R^2^/Q^2^) should necessarily perform well in virtual screening and vice versa. This is because: (1) Favorable R^2^/Q^2^ values typically characterize QSAR models operating within their applicability domains. However, in the context of virtual screening, such models are often expected to go beyond this domain. (2) Poor R^2^/Q^2^ values can still characterize models that are nevertheless able to successfully classify compounds into two group (e.g., active and inactive) as in fact done in virtual screening. Stated differently, a poor regressor is not necessarily a poor classifier.

With this in mind, the present work has two main goals: (1) Testing whether “standard” model evaluation metrics could inform on the performances of QSAR models in VS. (2) Developing a novel algorithm for the derivation of QSAR models (in the form of MLR equations) by directly optimizing the most common metric employed in VS, namely, enrichment.

With respect to the first goal, using MLR models derived for seven data sets by optimizing the model’s standard deviation, we have demonstrated a lack of correlation between the $R^{2}/Q_{F1/F2/F3}^{2}$ metrics and the number of active compounds retrieved/enrichment metric. This lack of correlation suggests that the performances of MLR models in the context of VS cannot be reliably predicted from their performances on a training set or even on a validation set. Thus, models with either favorable or poor R^2^/Q^2^ values performed both well and poorly when evaluated in the context of VS. A lack of correlation between an R^2^/Q^2^-like metric (the RMSD between experimental and predicted EC_50_ values) and enrichment was anecdotally observed by Mueller et al. in an artificial neural network (ANN)-based VS of mGluR5 potentiators [23]. For the M2 dataset, we have traced this lack of correlation to a potential incompatibility between test set compounds and the models’ applicability domain. We propose that this is but one example of a more general phenomenon, namely, that random compounds largely fall outside the applicability domain of the MLR models derived in this work. This suggestion is supported by the observed lack of correlation between the enrichment metric calculated for the training/validation sets and for the test sets.

With respect to the second goal, we have demonstrated, on the same datasets that models derived by optimizing an enrichment-like function (i.e., EOA models), provide results that are more consistent across training/validation/test sets. Furthermore, in the context of VS, almost all of these models performed either similar to or better than the MLR models, perhaps due to a larger applicability domain. Furthermore, when tested as binary classifiers, EOA models demonstrated superiority over models derived by the RF or SVM methods.

The presumed larger applicability domain of the EOA models, suggests that hits retrieved upon the application of such models to VS, may be more diverse than hits retrieved when VS is performed with “standard” MLR models. Since “standard” MLR models work well only within their (smaller) applicability domain, they will likely correctly predict the activities of only a small portion of the database, that portion which is structurally similar to the compounds used for their construction. Chemotype diversity of hit compounds is important in particular in early stages of drug development projects. Finally, while in the present work, MLR and EOA models were developed based on identical datasets (to allow for a fair comparison), the ability of EOA to use binary activity data is likely to translate into models covering larger parts of the chemical space. This is because available databases typically contain more compounds with binary than quantitative activity data. Such models will have larger applicability domains and consequently will again be able to retrieve hits that are more diverse. This however is also true for other classification-based models.

## 4. Methods

### 4.1. Datasets

Datasets containing experimentally measured ligand affinities data (*pKi* values or categorical values) for six protein targets belonging to the GPCR family, namely, 5-Hydroxytryptamine Receptor 2C (5HT_2C_), Muscarinic acetylcholine receptor M2 (M2), Histamine H1 receptor (H1), Muscarinic acetylcholine receptor M3 (M3), Dopamine receptor (D1), and Alpha-2C adrenergic receptor (Alpha_2C_) were retrieved from the ChEMBL [31] database. A seventh data set containing experimentally measured inhibition constants (*pKi* values) for inhibitors of the human Ether-à-go-go-Related Gene (hERG) anti-target was retrieved from Braga et al. [32].

All datasets considered in this work (including the ZINC compounds; see below) were first processed by Schrodinger’s LigPrep program [33] in order to obtain reliable conformations, tautomeric forms and protonation states. Next, each dataset was subjected to descriptors calculations by the Canvas program [34]. Overall, 760 descriptors were calculated and were subsequently pre-processed by removing constant, nearly constant (70%) and correlated ($r^{2}$ > 0.7) descriptors. These remaining descriptors were normalized using Z-Score normalization.

Next, compounds were assigned at random into training sets, validation sets and test sets as detailed below. Overall, training and validation sets were of similar sizes yet they varied in terms of the percentage of active vs. inactive compounds. Thus, some of the sets were balanced whereas others included many more inactive compounds than active compounds. This imbalance was introduced since we were interested in the application of the resulting equations to virtual screening where the main challenge is the identification of a small number of active compounds from within a set of random yet presumed to be largely inactive compounds. This imbalance was even more pronounced in the test sets, which were designed to mimic small-scale VS campaigns. For each model, two test sets were used. In the first (test set 1), the same set of active compounds used in the validation set was taken and combined with ~5000 decoys retrieved from the ZINC database [35]. Using the same set of active compounds for both validation and test was done for two reasons: (1) For some of the datasets the number of active compounds was too small to allow for spreading them across training, validation and test sets. For the sake of consistency, we decided to treat all datasets in the same manner. (2) Using the same set of active compounds in both validation and test sets, allowed us to hypothesize on reasons for differences in performances between the two sets as we have done. We note that the validation sets were not used in any way to train the models or to select best models and therefore the overlap in active compounds did not affect the performances of the models on the validation/test sets in any way. A second test set (test set 2) was constructed by taking all active compounds, which were not assigned to any of the previous sets and combining them with the same decoy compounds. For some targets, test set 2 only contained a very small number of active compounds.

### 4.2. Multiple Linear Regression (MLR)

In this work, we chose to compare our algorithm with Multiple Linear Regression (MLR) coupled with feature selection, which is a common method for deriving QSAR models. MLR models were built for each of the seven datasets considered in this work using the Canvas package [34]. Canvas builds MLR models by optimizing the regression standard deviation of the model in the space of the descriptors [36]. Training, validation and test sets for the seven targets were built as described above. For the 5HT_2C_, M2, H1, D1, M3, Alpha_2C_, and hERG datasets, active compounds were defined as having *pKi* values > 8.6, 8.5, 8.5, 8.01, 9.2, 8.0 and 10.5, respectively whereas inactive compounds were defined as having *pKi* values < 7.52, 5.56, 5.5, 5.78, 5.81, 6.12 and 7.52, respectively. We deliberately kept a gap between active and inactive compounds to avoid potential experimental errors. Reducing this gap is however beneficial since it will allow to construct models based on more data points. This will be dealt with in future work. Table 5 provides information on the datasets used for the derivation of MLR models.

For each set, MLR models were derived using 7, 10, and 13 descriptors. All parameters for model derivation were set to their default values except that the number of Monte Carlo (MC) steps used for the optimization was set to 10^6^. Models were derived from the training sets, validated on the validation sets and used to virtually screen the test sets.

Model performances on the training and validation sets were evaluated using $R^{2},$ $Q_{F1}^{2}$, $Q_{F2}^{2}$ and $Q_{F3}^{2}$ (Equations (1)–(4)):

(1) $$R^{2}=1-\frac{\sum_{i=1}^{N}{\left(y_{i}^{fit}-y_{i}\right)}^{2}}{\sum_{i=1}^{N}{\left(y_{i}-y_{mean}\right)}^{2}}\;\equiv 1-\frac{RSS}{SS}$$

(2) $$Q_{F1}^{2}=1-\frac{\sum_{i=1}^{N_{EXT}}{\left(y_{i}^{pred}-y_{i}\right)}^{2}}{\sum_{i=1}^{N_{EXT}}{\left(y_{i}-y_{mean-TR}\right)}^{2}}\;\equiv 1-\frac{PRESS}{SS_{EXT}\left(y_{mean-TR}\right)}$$

(3) $$Q_{F2}^{2}=1-\frac{\sum_{i=1}^{N_{EXT}}{\left(y_{i}^{pred}-y_{i}\right)}^{2}}{\sum_{i=1}^{N_{EXT}}{\left(y_{i}-y_{mean-EXT}\right)}^{2}}\;\equiv 1-\frac{PRESS}{SS_{EXT}\left(y_{mean-EXT}\right)}$$

(4) $$Q_{F3}^{2}=1-\frac{\left[\sum_{i=1}^{N_{EXT}}{\left(y_{i}^{pred}-y_{i}\right)}^{2}\right]/nEXT}{\left[\sum_{i=1}^{N_{EXT}}{\left(y_{i}-y_{mean-TR}\right)}^{2}\right]/nTR}\;\equiv 1-\frac{PRESS/nEXT}{SS_{EXT}\left(y_{mean-TR}\right)/nTR}$$

where in Equation (1), the summation runs over all training set compounds, $y_{i}^{fit}$ are the predicted (fitted) values, $y_{mean}$ are the mean activities, RSS is the residual sun of squares and SS is the total sum of squares. In Equation (2), the summation runs over all validation set compounds, $y_{i}^{pred}$ are the predicted values, $y_{mean-TR}$ are the mean activities for the training set compounds, PRESS is the predicted sum of squares and $SS_{EXT}\left(y_{mean-TR}\right)$ is the total sum of squares for the validation set calculated by the training set mean. In Equation (3), the mean is calculated from the validation set. In Equation (4) the *PRESS* is divided by the number of compounds of the validation set and the $SS_{EXT}\left(y_{mean-TR}\right)$ is divided by the number of compounds of the training set [14].

In addition, models’ performances on all sets were also evaluated by the number of active compounds found within the first *L* places of a list sorted according to the predicted *pKi* values, where *L* denotes the number of active compounds used in the training/validation/test sets. We note that the test sets were constructed to mimic small-scale virtual screening campaigns by having a relatively small number of known actives embedded in a much larger pool of random, yet presumed to be inactive compounds.

### 4.3. Random Forest (RF) and Support Vector Machine (SVM)

Random Forest (RF) is a classification technique which operates by deriving multiple decision trees and combining their predictions using a consensus approach [35]. In this work we have used the RF method as implemented in the Canvas package [34]. The ensemble model was generated based on 1000 trees. All other parameters were set to their default values. No restrictions were imposed on the number of descriptors used to construct the classifiers.

Support Vector Machine (SVM) is a supervised machine-learning algorithm that can be used either as a classifier or as a regressor. When used as a classifier, as done in the present work, SVM classifies compounds into two classes (e.g., active and inactive) by finding a hyperplane that maximizes the separation between the classes [37,38]. In the present work we use the SVM algorithm, specifically LIBSVM [39], as implemented in the WEKA package [40], with default parameters. No restrictions were imposed on the number of descriptors used to construct the classifiers.

For the purpose of deriving RF/SVM models, we have randomly selected from each of the seven parent datasets considered in this work eight subsets each consisting of 50 active compounds and 450 inactive compounds and used four of them as training sets and four as validation sets. Test sets 1 and 2 were constructed as described above. For the 5HT_2C_, M2, H1, hERG, M3, D1, and Alpha_2C_ datasets, active compounds were defined as having *pKi* values > 8.5, 9.0, 8.0, 9.52, 9.12, 8.0, and 8.1, respectively and compounds having *pKi* values < 4.5 or labeled as “inactive” populated the inactive compounds pool. For each subset, models were derived using 7, 10, and 13 descriptors for a total of 12 models. Table 6 provides information on the datasets used for the derivation of the RF/SVM models.

Models performances were first evaluated by counting the number of active compounds within the top *L* = 50 places of the ranked activity list and by calculating the corresponding enrichment value at the (L/M)×100 point of the library according to:

(5) $$Enrichment\left(\left(L/M\right)\times 100\right)=\frac{\%\;active\;compounds\;in\;L\;first\;places}{\%\;active\;compounds\;in\;library}$$

where (L/M)×100 determines the percentage of library at which enrichment is calculated (in this work, limited to a single point determined by the percentage of active compounds in the dataset). Performances were evaluated for training validation and test sets.

In addition, each model was characterized by the Matthew Correlation Coefficient (MCC) according to:

(6) $$MCC=\;\frac{TP\times TN-FP\times FN}{\sqrt{\left(TP+FP\right)\left(TP+FN\right)\left(TN+FP\right)\left(TN+FN\right)}}$$

where *TP*, *TN*, *FP*, and *FN* stand for true positives, true negatives, false positives and false negatives, respectively.

### 4.4. A Novel Algorithm for the Derivation of QSAR Equations (Enrichment Optimization Algorithm; EOA)

In this work, we present a novel algorithm for the derivation of QSAR equations suitable for virtual screening, based on the optimization of an enrichment-like objective function. We chose Monte Carlo/Simulated Annealing (MC/SA) as the optimization engine due to its generality and ease of implementation and since it is also the method implemented in Canvas’s MLR algorithm. Still, other optimization engines, for example, genetic algorithm or particle swarm optimization could also be used and may lead to faster convergence. The algorithm is composed of two phases, namely model derivation and model selection. The model derivation phase accepts as input a set of *L* active compounds embedded within a set of weakly active/inactive compounds (the algorithm can handle imbalanced sets with a ratio of active:weakly active/inactive ≤1:10) where each of the compounds is characterized by *N* molecular descriptors. It then maximizes the number of the *L* actives within the first *L* places of an ordered list of predicted activities generated as follows: At each step, a subset of *k* descriptors and *k* weights is selected and used to build a simple MLR model to predict the activities of all compounds. The activities are then sorted from highest to lowest and the number of known active compounds within the first *L* places of the resulting ordered list is counted. This number is then maximized, using a MC/SA procedure, in the space of the descriptors and weights. In this first implementation of the algorithm, the number of descriptors was held constant and only their identities and weights were changed. The specific steps are as follows:

1. Given a dataset of *M* compounds (of which *L* are active), characterized by *N* descriptors:
2. Select ${\{X_{i}\}}_{i=1,\;k}$ random descriptors.
3. Select ${\{C_{i}\}}_{i=1,\;k}$ random weights.
4. For each compound calculate a predictive activity value: $A_{j}=\overset{k}{\underset{i=1}{\sum }}X_{i}C_{i}$.
5. Sort ${\left\{A_{j}\right\}}_{j=1,M}$, from highest to lowest.
6. Count the number of known actives, within the first *L* places of the sorted list. Call this number $P_{1}$.
7. Optionally select new descriptors with new weights and/or modify the weights of the current descriptors so that $C_{i}^{new}=\;C_{i}^{old}\pm \Delta J$, where, ΔJ=random number between specific ranges.
8. Calculate $A_{j}^{new}=\overset{k}{\underset{i=1}{\sum }}X_{i}C_{i}^{new}$ or $A_{j}^{new}=\overset{k}{\underset{i=1}{\sum }}X_{i}^{new}C_{i}^{new}$
9. Sort {$A_{j}^{new}{\}}_{j=1,M}$, from highest to lowest.
10. Count the number of actives, within the first *L* places of the sorted list. Call the number $P_{2}$.
11. If $P_{2}>\;P_{1}$, accept and set: $P_{1}=\;P_{2}$; $X_{i}^{old}=$ $X_{i}^{new}$; $C_{i}^{old}=$ $C_{i}^{new}$.
12. If $P_{2}\leq \;P_{1},$ accept according to the Metropolis MC criterion:
13. A number, *r*, between 0 to 1 is generated randomly and the step is accepted if $r<e^{-\frac{\Delta E}{RT}}$, where $\Delta E=P_{1}-P_{2}$. When using MMC simulations to obtain the canonical ensemble, *R* is the gas constant and *T* is the absolute temperature. When using MMC as global optimizer as in the present case, *R* and *T* are constants with no physical meaning and their values simply determine the acceptance rate. In the present case, the term *RT* was linearly reduced in accord with the simulated annealing procedure.
14. If the step is rejected, keep the old values of the descriptors and weights.
15. Go back to step 7.
16. During the simulation process, keep the best value of *P*, *P_best_* and its associated descriptors and weights. If several solutions lead to the same value of *P_best_*, keep them all.

The above procedure is run until a pre-defined number of MC steps has been performed or until a model producing the highest possible value for the objective function (*L*) has been developed.

One potential drawback of using enrichment (as calculated in this work) as the objective function to be optimized is that the range of possible values is rather small. Thus, for a data set containing *L* active compounds, the objective function can be characterized by at most *L+1* values. For example, for *L* = 10, the objective function is limited to a value from within {0, 1, 2, 3, 4, 5, 6, 7, 8, 9, 10}. As a result, several QSAR equations may lead to the same final value of the objective function (*P_best_*) requiring the development of a post-processing ranking mechanism. Within the framework of the current algorithm, we did not attempt to rank different solutions that produced the highest possible value of the objective function (i.e., for which *P_best_ = L*) but simply let the procedure terminate if such a solution was obtained. Several mechanisms could be envisioned for ranking such solutions but would require numerical values for the activities of known actives (e.g., *Ki* or *IC_50_* values). This requirement will limit the scope of the method to cases where this information is available. However, for different solutions that produced the same, albeit not maximal value of the objective function, i.e., solutions that were able to rank *P_best_ < L* active compounds within the first *L* places in the ordered list, a post-processing ranking mechanism was implemented. To this end we examined active compounds that were ranked beyond the first *L* places and inactive compounds that were ranked within the first *L* places and preferred solutions where both types of compounds had ranks closer to *L*. To account for the skewness of the dataset (i.e., having more inactive than active compounds) the ranks of the active and inactive compounds were independently normalized using z-score. Specifically, the following steps were implemented:

1. Keep a list *{B}* of the *Z* best solutions having the best score, ${\left\{B_{l}\right\}}_{l=1,Z}=\;P_{best}$.
2. For each solution Z in *{B}* independently normalize the indices of the *L* active compounds and the *M-L* inactive compounds. Designate the normalized indices as *j’* and *j’’*, respectively.
3. For each solution *Z* in *{B}* calculate a score *S_Z_* as follows:
  - Sum the normalized *j’* indices for the active compounds with ranks > *L*, *S_active_ =* $\overset{M-L}{\underset{v=1}{\sum }}j\prime_{v}$.
  - Sum the normalized *j’’* indices for the inactive compounds with ranks < *L*, *S_inactive_ =* $\overset{L}{\underset{u=1}{\sum }}{j^{\prime \prime }}_{u}$.
  - Set Sz = S_active_ − S_inactive_
4. The solution with the lowest score is the best one and will be tested on the validation and test sets.

The above-described algorithm was implemented via a series of scripts programmed in Python version 3.6.

We have used the new algorithm to derive EOA models for all the datasets described in Table 5 and Table 6 using the same number of descriptors and the same compositions of training sets/validation sets/test sets used for the MLR (Section 4.2), RF and SVM (Section 4.3) analyses. For comparison with MLR, 15 models were derived for each dataset, consisting of five models for each the three sets of descriptors (7, 10, and 13 descriptors). The models differed by the random seeds used to initiate the MC optimization process. For comparison with RF/SVM, 60 models were derived for each dataset, consisting of five models for each of the seven training/validation/test sets and the three sets of descriptors. As for MLR, the models differed by the random seeds used to initiate the MC optimization process. The performances of all resulting models were evaluated by the number of active compounds found within the first *L* places of a list sorted according to the predicted *pKi* values, where *L* denotes the number of active compounds used in the training/validation/test sets. This number was converted into an enrichment factor using Equation (5). In the case of RF/SVM, models were also evaluated using MCC.

A typical MC/SA run consisted of 10^6^ MC steps. Simulated annealing was implemented by means of a saw tooth procedure whereby repeated annealing cycles where performed. In each cycle the *RT* term was linearly decreased from 0.3 to 0.01 or from 0.6 to 0.01 in 0.01 intervals, running 300 MC steps per interval. The range of values of the *RT* term were defined to maintain an acceptance rate of ~25–30% for the M2, H1, 5HT_2C_ and hERG sets, and ~45–60% for the D1, M3 and Alpha_2C_ sets.

All training/validation/test sets used in this work are provided in Tables S10–S16 in the Supplementary Materials.

## 5. Conclusions

The two main conclusions emerging from this work are the following: (1) QSAR models derived in order to be used for VS should be best evaluated using a metric that can reflect on their success in a VS campaign. This conclusion is a specific example of a more general principle suggesting that computational models in general, should be derived with their intended usage in mind. (2) Deriving QSAR models by directly optimizing an enrichment-based metric is a promising strategy for the development of QSAR models that could favorably be used as classifiers and for VS.

Clearly more work along several research lines including algorithmic improvements and application to additional datasets should be performed in order to quantify the extent of improvement in enrichment/diversity and consequently in VS performances, that could be achieved with the new algorithm. One drawback of the current EOA algorithm is that initiating model derivation with different random seeds leads to different final models with different performances, in particular on test sets, thereby complicating the selection of the model to be actually used for VS. This diversity in performances may suggest that the search in descriptors space for the best descriptors’ combinations did not fully converge calling for better optimizers. However, the overall good performances of all models on the training sets argues against this explanation. Another potential explanation is that the enrichment-based metric optimized by the EOA algorithm can accept only a small set of values (as explained in more details in the Methods section). This suggests that a range of QSAR models can yield excellent results for the training set yet perform sub-optimally on validation/test sets. This problem could be overcome by developing more complex, “values-rich” enrichment-based functions, for example by also including information about inactive compounds that should be pushed to the bottom of the ranked list. In addition, the availability of multiple QSAR models calls for a consensus approach for virtual screening. Finally, it will be important to test whether other model derivation techniques besides MLR, which might be able to generate models with better prediction statistics could also benefit from being evaluated by a VS-aware metric. Work along these lines is currently being performed in our laboratory.

## Figures and Tables

**Table 1:** Results of the Multiple Linear Regression (MLR) models.

Set	# Actives = *L*	# Descriptors	MC Steps	Train		Validation				Test1	Test2	
				$R^{2}$	# Actives Among *L* Top Places (Enrichment)	$Q_{F1}^{2}$	$Q_{F2}^{2}$	$Q_{F3}^{2}$	# Actives among *L* Top Places (Enrichment)	# Actives among *L* top Places (Enrichment)	# Actives = *L*	# Actives among *L* Top Places (Enrichment)
M2	50	7	10^6^	0.77	48 (2.6)	0.66	0.66	0.67	43 (2.4)	32 (66.4)	67	47 (54.5)
M2		10	10^6^	0.80	48 (2.6)	0.63	0.63	0.64	45 (2.5)	0 (0)		1 (1.2)
M2		13	10^6^	0.82	48 (2.6)	0.61	0.61	0.62	42 (2.3)	0 (0)		0 (0)
H1	50	7	10^6^	0.73	47 (2.5)	0.59	0.59	0.63	42 (2.3)	0 (0)	42	0 (0)
H1		10	10^6^	0.78	48 (2.6)	0.45	0.45	0.49	39 (2.1)	0 (0)		0 (0)
H1		13	10^6^	0.82	49 (2.6)	0.56	0.56	0.60	41 (2.2)	0 (0)		0 (0)
5HT_2C_	50	7	10^6^	0.58	43 (2.4)	0.14	0.14	0.18	32 (1.8)	10 (20.8)	58	21 (32.5)
5HT_2C_		10	10^6^	0.65	44 (2.5)	0.08	0.08	0.12	34 (1.9)	1 (2.1)		5 (7.7)
5HT_2C_		13	10^6^	0.70	45 (2.5)	−0.08	−0.08	−0.03	30 (1.7)	1 (2.1)		6 (9.3)
hERG	100	7	10^6^	0.34	67 (4.7)	0.28	0.28	0.16	64 (4.5)	87 (45.6)	26	21 (160.5)
hERG		10	10^6^	0.36	69 (4.8)	0.32	0.31	0.23	68 (4.8)	87 (45.6)		23 (175.8)
hERG		13	10^6^	0.39	71 (5.0)	0.33	0.33	0.24	72 (5.0)	91 (47.7)		22 (168.2)
M3	75	7	10^6^	0.85	74 (2.0)	0.66	0.66	0.68	68 (1.8)	0 (0)	4	0 (0)
M3		10	10^6^	0.89	74 (2.0)	0.68	0.68	0.70	67 (1.8)	0 (0)		0 (0)
M3		13	10^6^	0.91	75 (2.0)	0.73	0.73	0.75	70 (1.9)	0 (0)		0 (0)
D1	58	7	10^6^	0.83	57 (2.0)	0.81	0.81	0.80	56 (1.9)	20 (30.9)	20	2 (25.8)
D1		10	10^6^	0.86	57 (2.0)	0.77	0.77	0.75	57 (2.0)	0 (0)		0 (0)
D1		13	10^6^	0.88	58 (2.0)	0.74	0.74	0.72	56 (1.9)	0 (0)		0 (0)
Alpha_2C_	57	7	10^6^	0.77	53 (1.9)	0.77	0.77	0.77	56 (2.0)	33 (52.8)	1	0 (0)
Alpha_2C_		10	10^6^	0.80	53 (1.9)	0.70	0.70	0.70	55 (1.9)	26 (41.6)		0 (0)
Alpha_2C_		13	10^6^	0.83	54 (1.9)	0.71	0.71	0.72	53 (1.9)	29 (46.4)		0 (0)

**Table 2:** Best (out of five repeats, based on the performances on the test sets) results obtained for the seven datasets using Enrichment Optimizer Algorithm (EOA). A compilation of all the results is provided in Tables S2–S8 (first sheet in each table) in the Supplementary Materials. Red, yellow and green coloring represent cases where EOA performances on test sets are poorer than, similar to or better than the corresponding Multiple Linear Regression (MLR) models presented in Table 1.

Set	# Descriptors	# Actives = *L*	MC Steps	# Actives among *L* Top Places in Best Model (Enrichment)			Test2	
				Train	Validation	Test1	# Actives = *L*	# Actives among *L* Top Places in Best Model (Enrichment)
M2	7	50	10^6^	47 (2.5)	40 (2.1)	40 (83.1)	67	56 (65.0)
M2	10	50	10^6^	47 (2.5)	44 (2.4)	39 (81.0)		54 (62.6)
M2	13	50	10^6^	47 (2.5)	42 (2.3)	38 (78.9)		54 (62.6)
H1	7	50	10^6^	48 (2.6)	37 (2.1)	31 (64.4)	42	23 (67.6)
H1	10	50	10^6^	49 (2.6)	42 (2.3)	32 (66.4)		24 (70.5)
H1	13	50	10^6^	48 (2.6)	38 (2.0)	32 (66.4)		22 (64.6)
5HT_2C_	7	50	10^6^	45 (2.5)	34 (1.9)	0 (0)	58	6 (9.3)
5HT_2C_	10	50	10^6^	45 (2.5)	32 (1.8)	1 (2.1)		8 (12.4)
5HT_2C_	13	50	10^6^	47 (2.6)	33 (1.8)	0 (0)		0 (0)
hERG	7	100	10^6^	67 (4.7)	60 (4.2)	86 (45.1)	26	22 (168.2)
hERG	10	100	10^6^	75 (5.3)	61 (4.3)	89 (46.6)		23 (175.8)
hERG	13	100	10^6^	77 (5.4)	59 (4.1)	87 (45.6)		23 (175.8)
M3	7	75	10^6^	74 (2.0)	65 (1.7)	49 (45.4)	4	3 (964.7)
M3	10	75	10^6^	74 (2.0)	67 (1.8)	0 (0)		0 (0)
M3	13	75	10^6^	74 (2.0)	70 (1.9)	57 (52.9)		3 (964.7)
D1	7	58	10^6^	56 (1.9)	54 (1.9)	29 (44.8)	20	11 (141.9)
D1	10	58	10^6^	57 (2.0)	55 (1.9)	20 (30.9)		1 (12.9) *
D1	13	58	10^6^	57 (2.0)	54 (1.9)	41 (63.4)		17 (219.3)
Alpha_2C_	7	57	10^6^	49 (1.7)	47 (1.6)	25 (40.0)	1	0 (0)
Alpha_2C_	10	57	10^6^	49 (1.7)	45 (1.6)	25 (40.0)		0 (0)
Alpha_2C_	13	57	10^6^	56 (2.0)	52 (1.8)	15 (24.0)		0 (0)

* This is the only case where a different model gave better results on Test2. The statistics of the model in terms of the number of active compounds retrieved within the first L place (enrichment) are: Training: 57 (2.0); Validation: 53 (1.8); Test1: 14 (21.6); Test2: 3 (38.7).

**Table 3:** A comparison of the performances of Enrichment Optimizer Algorithm (EOA) models with those of Random Forest (RF) and Support Vector Machine (SVM) for the seven datasets considered in this work. For EOA we provide the results obtained with the best models as determined according to performances on test set 1. A complete listing of the results obtained with all models is provided in Tables S2–S8 (2nd–5th sheets) of the Supplementary Materials. Each cell contains the number of active compounds found within the first *L* places and in parenthesis, the enrichment calculated according to Equation (5) and the Matthews Correlation Coefficient (MCC). Green coloring represent the best models (as judged be performances on the test set) from within EOA, RF and SVM.

Set	Run	# Actives among *L* Top Places (Enrichment; MCC)								
		EOA			RF			SVM		
		Train	Validation	Test 1	Train	Validation	Test 1	Train	Validation	Test 1
M2	1	46 (9.2; 0.91)	41 (8.2; 0.80)	50 (103.8; 1.00)	42 (8.4; 0.88)	39 (7.8; 0.77)	39 (81.0; 0.88)	43 (8.6; 0.92)	36 (7.2; 0.84)	36 (74.8; 0.85)
	2	46 (9.2; 0.91)	40 (8.0; 0.78)	48 (99.7; 0.96)	42 (8.4; 0.83)	37 (7.4; 0.73)	37 (76.8; 0.86)	42 (8.4; 0.83)	37 (7.4; 0.84)	37 (76.8; 0.86)
	3	45 (9.0; 0.89)	41 (8.2; 0.80)	47 (97.6; 0.94)	43 (8.6; 0.86)	42 (8.4; 0.79)	42 (87.2; 0.92)	41 (8.2; 0.84)	37 (7.4; 0.73)	37 (76.8; 0.86)
	4	44 (8.8; 0.87)	42 (8.4; 0.82)	48 (99.7; 0.96)	40 (8.0; 0.81)	40 (8.0; 0.80)	40 (83.1; 0.89)	41 (8.2; 0.82)	38 (7.6; 0.77)	38 (78.9; 0.87)
H1	1	44 (8.8; 0.87)	36 (7.2; 0.69)	38 (78.9; 0.76)	46 (9.2; 0.92)	29 (5.8; 0.61)	29 (60.2; 0.73)	41 (8.2; 0.88)	33 (6.6; 0.76)	33 (68.5; 0.81)
	2	42 (8.4; 0.82)	31 (6.2; 0.58)	47 (97.6; 0.94)	47 (9.4; 0.91)	33 (6.6; 0.67)	33 (68.5; 0.78)	40 (8.0; 0.85)	34 (6.8; 0.78)	34 (70.6; 0.82)
	3	38 (7.6; 0.73)	34 (6.8; 0.64)	42 (87.2; 0.84)	47 (9.4; 0.88)	39 (7.8; 0.77)	39 (81.0; 0.70)	39 (7.8; 0.86)	31 (6.2; 0.73)	31 (64.4; 0.79)
	4	43 (8.6; 0.84)	29 (5.8; 0.53)	39 (81.0; 0.78)	41 (8.2; 0.82)	27 (5.4; 0.46)	27 (56.1; 0.52)	39 (7.8; 0.79)	27 (5.4; 0.7)	27 (56.1; 0.73)
5HT_2C_	1	50 (10.0; 1.00)	50 (10.0; 1.00)	50 (103.8; 1.00)	50 (10.0; 0.98)	50 (10.0; 0.98)	50 (103.8; 1.00)	50 (10.0; 1.00)	48 (9.6; 0.98)	48 (99.7; 0.98)
	2	50 (10.0; 1.00)	50 (10.0; 1.00)	50 (103.8; 1.00)	50 (10.0; 0.98)	50 (10.0; 0.99)	50 (103.8; 1.00)	50 (10.0; 1.00)	50 (10.0; 1.00)	50 (103.8; 1.00)
	3	50 (10.0; 1.00)	50 (10.0; 1.00)	50 (103.8; 1.00)	50 (10.0; 0.99)	50 (10.0; 0.96)	50 (103.8; 1.00)	50 (10.0; 1.00)	50 (10.0; 1.00)	50 (103.8; 1.00)
	4	50 (10.0; 1.00)	50 (10.0; 1.00)	50 (103.8; 1.00)	50 (10.0; 0.98)	50 (10.0; 0.99)	50 (103.8; 1.00)	50 (10.0; 1.00)	50 (10.0; 1.00)	50 (103.8; 1.00)
hERG	1	41 (8.2; 0.80)	29 (5.8; 0.53)	42 (87.2; 0.84)	39 (7.8; 0.83)	23 (4.6; 0.41)	23 (47.8; 0.31)	35 (7.0; 0.81)	19 (3.8; 0.55)	19 (39.5; 0.61)
	2	42 (8.4; 0.82)	22 (4.4; 0.38)	38 (78.9; 0.76)	42 (8.4; 0.90)	26 (5.2; 0.56)	26 (54.0; 0.33)	38 (7.6; 0.84)	19 (3.8; 0.52)	19 (39.5; 0.61)
	3	39 (7.8; 0.76)	23 (4.6; 0.40)	45 (93.4; 0.90)	35 (7.0; 0.77)	32 (6.4; 0.68)	32 (66.4; 0.64)	31 (6.2; 0.77)	22 (4.4; 0.63)	22 (45.7; 0.66)
	4	38 (7.6; 0.73)	29 (5.8; 0.53)	47 (97.6; 0.94)	34 (6.8; 0.78)	19 (3.8; 0.49)	19 (39.5; 0.55)	33 (6.6; 0.8)	18 (3.6; 0.56)	18 (37.4; 0.60)
D1	1	42 (8.4; 0.82)	37 (7.4; 0.71)	44 (91.4; 0.88)	42 (8.4; 0.86)	33 (6.6; 0.71)	33 (68.5; 0.69)	45 (9.0; 0.94)	44 (8.8; 0.84)	44 (91.4; 0.94)
	2	42 (8.4; 0.82)	33 (6.6; 0.62)	46 (95.5; 0.92)	50 (10;.0 0.97)	31 (6.2; 0.71)	31 (64.4; 0.55)	44 (8.8; 0.91)	35 (7.0; 0.81)	35 (72.7; 0.84)
	3	41 (8.2; 0.80)	41 (8.2; 0.80)	45 (93.4; 0.90)	48 (9.6; 0.93)	37 (7.4; 0.68)	37 (76.8; 0.71)	46 (9.2; 0.94)	36 (7.2; 0.79)	36 (74.8; 0.85)
	4	39 (7.8; 0.76)	37 (7.4; 0.71)	45 (93.4; 0.90)	48 (9.6; 0.98)	40 (8.0; 0.82)	40 (83.1; 0.87)	42 (8.4; 0.91)	37 (7.4; 0.80)	37 (76.8; 0.86)
M3	1	44 (8.8; 0.87)	42 (8.4; 0.82)	49 (101.7 0.98)	50 (10.0; 0.98)	50 (10.0; 0.96)	50 (103.8; 1.00)	45 (9.0; 0.94)	45 (9.0; 0.92)	45 (93.4; 0.95)
	2	48 (9.6; 0.96)	44 (8.8; 0.87)	48 (99.7; 0.96)	38 (7.6; 0.84)	35 (7.0; 0.78)	35 (72.7; 0.84)	40 (8.0; 0.88)	35 (7.0; 0.82)	35 (72.7; 0.84)
	3	44 (8.8; 0.87)	41 (8.2; 0.80)	48 (99.7; 0.96)	41 (8.2; 0.88)	32 (6.4; 0.78)	32 (66.4; 0.80)	41 (8.2; 0.9)	33 (6.6; 0.80)	33 (68.5; 0.81)
	4	48 (9.6; 0.96)	44 (8.8; 0.87)	44 (91.4; 0.88)	44 (8.8; 0.92)	37 (7.4; 0.84)	37 (76.8; 0.86)	42 (8.4; 0.91)	37 (7.4; 0.85)	37 (76.8; 0.86)
Alpha_2C_	1	40 (8.0; 0.78)	34 (6.8; 0.64)	37 (76.8; 0.74)	42 (8.4; 0.86)	35 (7.0; 0.72)	35 (72.7; 0.84)	37 (7.4; 0.85)	33 (6.6; 0.77)	33 (68.5; 0.81)
	2	43 (8.6; 0.84)	29 (5.8; 0.53)	44 (91.4; 0.88)	42 (8.4; 0.87)	38 (7.6; 0.78)	38 (78.9; 0.87)	39 (7.8; 0.86)	34 (6.8; 0.78)	34 (70.6; 0.82)
	3	43 (8.6; 0.84)	35 (7.0; 0.67)	43 (89.3; 0.86)	41 (8.2; 0.85)	38 (7.6; 0.75)	38 (78.9; 0.79)	42 (8.4; 0.91)	34 (6.8; 0.76)	34 (70.6; 0.82)
	4	40 (8.0; 0.78)	37 (7.4; 0.71)	42 (87.2; 0.84)	39 (7.8; 0.81)	40 (8.0; 0.81)	40 (83.1; 0.89)	44 (8.8; 0.93)	41 (8.2; 0.82)	41 (85.1; 0.90)

**Table 4:** The same as Table 3 but the best Enrichment Optimizer Algorithm (EOA) models were selected based on their performances on test set 2. Asterisks (*) denote cases where the same model performed best for both test set 1 and test set 2.

Set	Run	# Actives among *L* Top Places (Enrichment; MCC)								
		EOA			RF			SVM		
		Train	Validation	Test 2	Train	Validation	Test 2	Train	Validation	Test 2
M2	1 *	46 (9.2; 0.91)	41 (8.2; 0.80)	6 (857.8; 1.00)	42 (8.4; 0.88)	39 (7.8; 0.77)	5 (714.9; 0.91)	43 (8.6; 0.92)	36 (7.2; 0.84)	5 (714.9; 0.91)
	2	47 (9.4; 0.93)	37 (7.4; 0.71)	6 (857.8; 1.00)	42 (8.4; 0.83)	37 (7.4; 0.73)	5 (714.9; 0.91)	42 (8.4; 0.83)	37 (7.4; 0.84)	5 (714.9; 0.91)
	3	49 (9.8; 0.98)	42 (8.4; 0.82)	5 (714.9; 0.83)	43 (8.6; 0.86)	42 (8.4; 0.79)	1 (143; 0.41)	41 (8.2; 0.84)	37 (7.4; 0.73)	2 (285.9; 0.58)
	4 *	44 (8.8; 0.87)	42 (8.4; 0.82)	5 (714.9; 0.83)	40 (8.0; 0.81)	40 (8.0; 0.80)	5 (714.9; 0.91)	41 (8.2; 0.82)	38 (7.6; 0.77)	5 (714.9; 0.91)
H1	1	46 (9.2; 0.91)	36 (7.2; 0.69)	115 (32.8; 0.84)	46 (9.2; 0.92)	29 (5.8; 0.61)	84 (24; 0.77)	41 (8.2; 0.88)	33 (6.6; 0.76)	81 (23.1; 0.77)
	2 *	42 (8.4; 0.82)	31 (6.2; 0.58)	128 (34.5; 0.91)	47 (9.4; 0.91)	33 (6.6; 0.67)	85 (22.9; 0.76)	40 (8.0; 0.85)	34 (6.8; 0.78)	81 (21.8; 0.76)
	3 *	38 (7.6; 0.73)	34 (6.8; 0.64)	115 (34.3; 0.86)	47 (9.4; 0.88)	39 (7.8; 0.77)	88 (26.2; 0.72)	39 (7.8; 0.86)	31 (6.2; 0.73)	71 (21.2; 0.73)
	4 *	43 (8.6; 0.84)	29 (5.8; 0.53)	120 (33.3; 0.87)	41 (8.2; 0.82)	27 (5.4; 0.46)	90 (25.0; 0.70)	39 (7.8; 0.79)	27 (5.4; 0.70)	97 (26.9; 0.84)
5HT_2C_	1 *	50 (10.0; 1.00)	50 (10.0; 1.00)	98 (53.5; 1.00)	50 (10.0; 0.98)	50 (10.0; 0.98)	98 (53.5; 1.00)	50 (10.0; 1.00)	48 (9.6; 0.98)	97 (52.9; 0.99)
	2 *	50 (10.0; 1.00)	50 (10.0; 1.00)	97 (54.0; 1.00)	50 (10.0; 0.98)	50 (10.0; 0.99)	97 (54; 1.00)	50 (10.0; 1.00)	50 (10.0; 1.00)	97 (54.0; 1.00)
	3 *	50 (10.0; 1.00)	49 (9.8; 0.98)	98 (53.5; 1.00)	50 (10.0; 0.99)	50 (10.0; 0.96)	98 (53.5; 1.00)	50 (10.0; 1.00)	50 (10.0; 1.00)	98 (53.5; 1.00)
	4 *	50 (10.0; 1.00)	50 (10.0; 1.00)	98 (53.5; 1.00)	50 (10.0; 0.98)	50 (10.0; 0.99)	98 (53.5; 1.00)	50 (10.0; 1.00)	50 (10.0; 1.00)	98 (53.5; 1.00)
hERG	1 *	41 (8.2; 0.80)	29 (5.8; 0.53)	112 (37.2; 0.89)	39 (7.8; 0.83)	23 (4.6; 0.41)	65 (21.6; 0.47)	35 (7.0; 0.81)	19 (3.8; 0.55)	62 (20.6; 0.70)
	2 *	42 (8.4; 0.82)	22 (4.4; 0.38)	110 (78.9; 0.87)	42 (8.4; 0.90)	26 (5.2; 0.56)	72 (23.9; 0.49)	38 (7.6; 0.84)	19 (3.8; 0.52)	51 (16.9; 0.63)
	3	37 (7.4; 0.71)	28 (5.6; 0.51)	103 (34.2; 0.81)	35 (7.0; 0.77)	32 (6.4; 0.68)	70 (23.2; 0.66)	31 (6.2; 0.77)	22 (4.4; 0.63)	61 (20.2; 0.69)
	4	42 (8.4; 0.82)	32 (6.4; 0.6)	111 (36.8; 0.88)	34 (6.8; 0.78)	19 (3.8; 0.49)	42 (13.9; 0.54)	33 (6.6; 0.80)	18 (3.6; 0.56)	47 (15.6; 0.61)
D1	1 *	42 (8.4; 0.82)	37 (7.4; 0.71)	40 (102.4; 0.89)	42 (8.4; 0.86)	33 (6.6; 0.71)	35 (89.6; 0.75)	45 (9.0; 0.94)	44 (8.8; 0.84)	38 (97.3; 0.92)
	2	42 (8.4; 0.82)	37 (7.4; 0.71)	41 (105; 0.91)	50 (10.0; 0.97)	31 (6.2; 0.71)	35 (89.6; 0.64)	44 (8.8; 0.91)	35 (7.0; 0.81)	35 (89.6; 0.88)
	3	41 (8.2; 0.80)	37 (7.4; 0.71)	38 (93.2; 0.82)	48 (9.6; 0.93)	37 (7.4; 0.68)	39 (95.6; 0.77)	46 (9.2; 0.94)	36 (7.2; 0.79)	35 (85.8; 0.87)
	4	40 (8.0; 0.78)	37 (7.4; 0.71)	43 (105.4; 0.93)	48 (9.6; 0.98)	40 (8.0; 0.82)	44 (107.9; 0.96)	42 (8.4; 0.91)	37 (7.4; 0.80)	39 (95.6; 0.92)
M3	1 *	44 (8.8; 0.87)	42 (8.4; 0.82)	65 (77.7; 0.98)	50 (10.0; 0.98)	50 (10.0; 0.96)	66 (78.9; 1.00)	45 (9.0; 0.94)	45 (9.0; 0.92)	55 (65.7; 0.91)
	2 *	48 (9.6; 0.96)	44 (8.8; 0.87)	66 (78.9; 1.00)	38 (7.6; 0.84)	35 (7.0; 0.78)	47 (56.2; 0.84)	40 (8.0; 0.88)	35 (7.0; 0.82)	48 (57.4; 0.85)
	3 *	44 (8.8; 0.87)	41 (8.2; 0.8)	64 (76.5; 0.97)	41 (8.2; 0.88)	32 (6.4; 0.78)	40 (47.8; 0.78)	41 (8.2; 0.90)	33 (6.6; 0.80)	43 (51.4; 0.81)
	4	44 (8.8; 0.87)	41 (8.2; 0.8)	61 (72.9; 0.92)	44 (8.8; 0.92)	37 (7.4; 0.84)	47 (56.2; 0.84)	42 (8.4; 0.91)	37 (7.4; 0.85)	46 (55; 0.83)
Alpha_2C_	1 *	40 (8.0; 0.78)	34 (6.8; 0.64)	6 (255.5; 0.54)	42 (8.4; 0.86)	35 (7.0; 0.72)	8 (340.6; 0.85)	37 (7.4; 0.85)	33 (6.6; 0.77)	7 (298.0; 0.80)
	2 *	43 (8.6; 0.84)	29 (5.8; 0.53)	9 (383.2; 0.82)	42 (8.4; 0.87)	38 (7.6; 0.78)	9 (383.2; 0.90)	39 (7.8; 0.86)	34 (6.8; 0.78)	8 (340.6; 0.85)
	3 *	43 (8.6; 0.84)	35 (7.0; 0.67)	8 (340.6; 0.73)	41 (8.2; 0.85)	38 (7.6; 0.75)	7 (298.1; 0.54)	42 (8.4; 0.91)	34 (6.8; 0.76)	7 (298.0; 0.80)
	4 *	40 (8.0; 0.78)	37 (7.4; 0.71)	6 (482.7; 0.75)	39 (7.8; 0.81)	40 (8.0; 0.81)	5 (402.3; 0.79)	44 (8.8; 0.93)	41 (8.2; 0.82)	6 (482.7; 0.87)

**Table 5:** Description of the seven datasets used for the derivation of Multiple Linear Regression (MLR) models. The “Maximal Enrichment” column provides the maximal possible enrichment at *L*, attainable for the data set for a comparison with the enrichment values provided in Table 1 and Table 2.

Dataset	# Descriptors	Training Set			Validation Set			Test Set 1			Test Set 2		
		# Actives	# Inactives	Maximal Enrichment	# Actives	# Inactives	Maximal Enrichment	# Actives	# Random	Maximal Enrichment	# Actives	# Random	Maximal Enrichment
5HT_2C_	7, 10, 13	50	87	2.7	50	87	2.7	50	5141	103.8	67	5141	77.7
M2	7, 10, 13	50	84	2.7	50	84	2.7	50	5141	103.8	42	5141	123.4
H1	7, 10, 13	50	90	2.8	50	90	2.8	50	5141	103.8	58	5141	89.6
hERG	7, 10, 13	100	600	7.0	100	600	7.0	100	5141	52.4	26	5141	198.7
M3	7, 10, 13	75	75	2.0	75	75	2.0	75	5141	69.5	4	5141	1286.3
D1	7, 10, 13	58	58	2.0	58	58	2.0	58	5141	89.6	20	5141	258.1
Alpha_2C_	7, 10, 13	57	57	2.0	57	57	2.0	57	5141	91.2	1	5141	5142.0

**Table 6:** Description of the four datasets used for the derivation of Random Forest (RF) and Support Vector Machine (SVM) models. The “Maximal Enrichment” column provides the maximal possible enrichment at *L*, attainable for the data set for a comparison with the enrichment values provided in Table 3 and Table 4.

Dataset	# Descriptors	Training Set			Validation Set			Test Set 1			Test Set 2		
		# Actives	# Inactives	Maximal Enrichment	# Actives	# Inactives	Maximal Enrichment	# Actives	# Inactives	Maximal Enrichment	# Actives	# Inactives	Maximal Enrichment
5HT_2C_	7, 10, 13	50	450	10.0	50	450	10.0	50	5141	103.8	97–98 *	5141	53.4–54.0
M2	7, 10, 13	50	450	10.0	50	450	10.0	50	5141	103.8	6	5141	857.8
H1	7, 10, 13	50	450	10.0	50	450	10.0	50	5141	103.8	133–140 *	5141	37.7–39.7
hERG	7, 10, 13	50	450	10.0	50	450	10.0	50	5141	103.8	126	5141	41.8
M3	7, 10, 13	50	450	10.0	50	450	10.0	50	5141	103.8	66	5141	78.9
D1	7, 10, 13	50	450	10.0	50	450	10.0	50	5141	103.8	45–46 *	5141	112.8–115.2
Alpha_2C_	7, 10, 13	50	450	10.0	50	450	10.0	50	5141	103.8	8–11 *	5141	468.4–643.6

* Different sets have slightly different numbers of active compounds.

## Supplementary Materials

The following are available online at https://www.mdpi.com/1422-0067/21/21/7828/s1.
